# The Effect of Chinese Herbal Medicine on Albuminuria Levels in Patients with Diabetic Nephropathy: A Systematic Review and Meta-Analysis

**DOI:** 10.1155/2013/937549

**Published:** 2013-08-26

**Authors:** Ya Xiao, Yanyan Liu, Keqiang Yu, Lin Zhou, Jianlu Bi, Jingru Cheng, Fei Li, Ren Luo, Xiaoshan Zhao

**Affiliations:** ^1^Department of Traditional Chinese Medicine, Nanfang Hospital, Southern Medical University, Guangzhou, Guangdong 510515, China; ^2^School of Traditional Chinese Medicine, Southern Medical University, Guangzhou, Guangdong 510515, China; ^3^Endocrinology Department, Nanfang Hospital, Southern Medical University, Guangzhou, Guangdong 510515, China

## Abstract

To evaluate the effect of Chinese herbal medicine (CHM) on albuminuria levels in patients with diabetic nephropathy (DN), we performed comprehensive searches on Medline database, Cochrane Library, CNKI database, CBM database, Wanfang database, and VIP database up to December 2012. A total of 29 trials including 2440 participants with DN met the selection criteria. CHM was tested to be more effective in reducing urinary albumin excretion rate (UAER) (MD −82.95 **μ**g/min, [−138.64, −27.26]) and proteinuria (MD −565.99 mg/24 h, [−892.41, −239.57]) compared with placebo. CHM had a greater beneficial effect on reduction of UAER (MD −13.41 **μ**g/min, [−20.63, −6.19]) and proteinuria (MD −87.48 mg/24 h, [−142.90, −32.06]) compared with angiotensin converting enzyme inhibitors (ACEI) or angiotensin receptor blockers (ARB). Combination therapy with CHM and ACEI/ARB showed significant improvement in UAER (MD −28.18 **μ**g/min, [−44.4, −11.97]), urinary albumin-creatinine ratio (MD −347.00, [−410.61, −283.39]), protein-creatinine ratio (MD −2.49, [−4.02, −0.96]), and proteinuria (MD −26.60 mg/24 h, [−26.73, −26.47]) compared with ACEI/ARB alone. No serious adverse events were reported. CHM seems to be an effective and safe therapy option to treat proteinuric patients with DN, suggesting that further study of CHM in the treatment of DN is warranted in rigorously designed, multicentre, large-scale trials with higher quality worldwide.

## 1. Introduction

Diabetic nephropathy (DN), defined as the presence of micro- or macroalbuminuria in patients with diabetes, is the most common cause of end-stage renal disease (ESRD) across the world [1]. The prevalence of micro- and macroalbuminuria in patients with diabetes is as high as 37–40% in western countries and 57.4–59.8% in Asian countries [2–4]. Albuminuria is a well-established risk factor for cardiovascular disease and is also associated with ESRD [5, 6]. Persistent albuminuria has toxic effect on tubular epithelial cells, causing tubulointerstitial inflammation and subsequent interstitial fibrosis. Angiotensin converting enzyme inhibitors (ACEI) and angiotensin receptor blockers (ARB) have been demonstrated to reduce albuminuria and delay the progression of DN by inhibition of renin-angiotensin system (RAS) and have become the standard of care for albuminuric patients [7, 8]. Despite the renoprotective effects of ACEI and ARB, diabetic nephropathy progresses to ESRD in a large proportion of patients [9]. This indicates that in addition to the RAS, other pathways are involved in the pathogenesis of DN. Chinese herbal medicine (CHM), which can produce a potential effect of multitarget therapy and block these pathways, seems appropriate in the treatment of DN caused by multiple factors [10].

In traditional Chinese medicine, diabetic nephropathy is considered nearly equivalent to the term “Xiao Ke Bing,” which has been described in the “Yellow Emperor's Medicine Classic” (Chinese name in pinyin “Huang Di Nei Jing”) more than 2000 years ago. Bawei Dihuang wan, originated from the “The Synopsis of Prescriptions of the Golden Chamber” in the Eastern Han Dynasty, is a famous Chinese herbal formula that has been used for a long time in the treatment of DN. In recent years, more and more herbal products are thought to be effective in reducing urinary protein in patients with DN. A number of randomised controlled trials (RCTs) have suggested that CHM alone or combined with ACEI/ARB has therapeutic potential in the treatment of DN in terms of reducing urinary albumin excretion, ameliorating proteinuria, and symptom improvement [11]. How about the effect of CHM on albuminuria alone or in combination with ACEI/ARB as compared to ACEI/ARB? With a view to answering the question, the systematic review of randomized controlled trials evaluates the effects and safety of CHM on albuminuria in patients with DN.

## 2. Methods

### 2.1. Search Strategy

A comprehensive literature search was performed using Medline database (1989 to December 2012), Cochrane Library (1993 to December 2012), CNKI database (1979 to December 2012), Chinese Biomedical Literature database (1990 to December 2012), Wanfang database (1982 to December 2012), and VIP database (1989 to December 2012). Keywords for searching included diabetes or diabetic, nephropathy, kidney disease, traditional Chinese medicine, herbal-medicine, alternative-medicine, complementary-medicine, plants, herbs, and phytotherapy. The search was restricted to studies carried out in humans. No limit was placed on language. Manual searches of conference compilations supplemented electronic searches.

### 2.2. Study Selection

Studies were considered to be eligible for inclusion if they met all of the following criteria. (i) Patients included in the study were diagnosed with type 2 diabetes mellitus complicated with kidney disease, regardless of the stage of the DN (microalbuminuria defined as urine albumin excretion rate (UAER) of 20–200 *μ*g/min, or macroalbuminuria defined as UAER >200 *μ*g/min). (ii) The study was performed as a randomized controlled trial (RCT) describing a correct randomization procedure. Trials which used a clearly inappropriate method of randomization (e.g., open alternation) were excluded. (iii) The intervention of CHM included extract from herbs, single herbs, Chinese patent medicines, or a compound of herbs that was prescribed (individualized treatment) by Chinese practitioner. The control intervention included placebo or ACEI/ARB. Hypoglycemic therapy was used as a cointervention in both of the arms, including oral hypoglycemic drugs, insulin, and exercise. (iv) Outcomes included at least one of the following: urine albumin excretion rate, proteinuria, urinary albumin-creatinine ratio, or urinary protein-creatinine ratio. 

### 2.3. Data Extraction

Two researchers independently extracted data, including study design, randomization, blinding and subject characteristics (e.g., age, sex, sample size, and albuminuria stage), and duration of treatment. Disagreements were resolved after discussion with other investigators. 

### 2.4. Data Analysis

Meta-analysis was carried out using Review Manager software (version 5.1), provided by the Cochrane Collaboration. The mean change in each study end point from baseline was treated as a continuous variable. Continuous data were presented as mean difference (MD), with 95% confidence interval (CI). The chi-squared test for heterogeneity was performed, and heterogeneity was presented as significant when *I* ^2^ is over 50% or *P* < 0.1. Random effect model was used for the meta-analysis if there was significant heterogeneity, and fixed effect model was used when the heterogeneity was not significant. 

## 3. Results

### 3.1. Search Results

**Figure 1:**
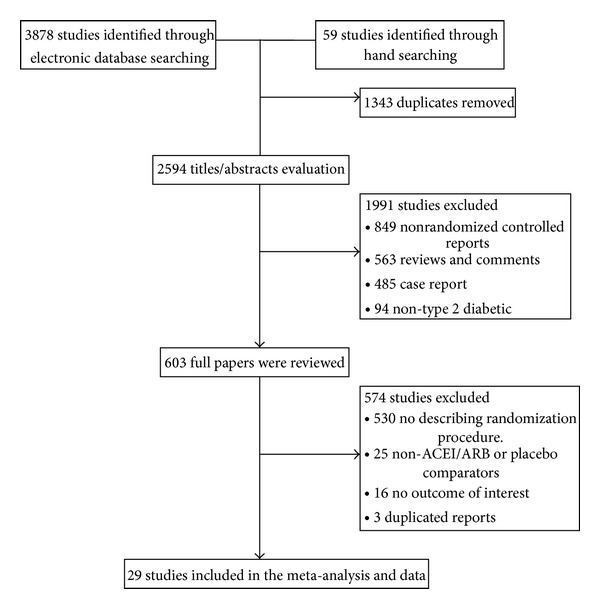
Flow chart of study selection process.

A total of 3937 publications were identified by both computer search and manual search of cited references. Of these, 1343 articles were determined to be duplicated. The remaining 2594 reports were retrieved in full text, of which 1991 were excluded on review of the titles and abstracts. After further reading, we excluded 530 for not describing randomization procedure, 25 non-ACEI/ARB or placebo comparators, 16 no outcome of interest, and 3 duplicated reports. Finally, a total of 29 studies were included in the meta-analysis.  Figure 1 is a flow chart of study selection process. 

### 3.2. Characteristics and Methodological Quality of Included Trials

All 29 publications included were of a randomization procedure generated by a random number table or computer [12–40]. Twenty-seven studies were published in Chinese and the other two in English. Numbers of participants of the individual studies varied from 40 to 409 with a total of 2440 participants included in this paper (Table 1). The majority duration of treatment varied from one month to three months. 

The Jadad scale is a 5-point scale for assessing the quality of RCTs in which three points or more indicate superior quality [41]. Of the 29 RCTs, 11 trials were of superior quality according to the Jadad score (≥3 points) [12, 15, 17, 21, 24, 25, 32, 34, 38–40]. All studies described a correct randomization procedure, but only one of them mentioned allocation concealment [39]. Three out of 29 studies described blinding of participants [12, 39, 40]. Ten trials reported the dropouts information and mentioned follow-up, but this dropouts were not captured in the analysis [12, 15, 17, 21, 24, 25, 32, 34, 38, 39]. Among all trials, the characteristics of participants in different treatment groups were similar at baseline (age, sex, race, and disease course). 

### 3.3. Analysis of Chinese Herbal Medicine

A total of 84 different kinds of herbs were included in 29 herbal preparations for treatment of DN. In Table 2, we listed the 14 herbs that were included most frequently in the 29 herbal preparations. For example, the herb used most often, *Astragalus membranaceus* (Huang Qi), was used 22 times in 29 different herbal preparations; the herb used second frequently, *Salvia miltiorrhiza* (Dan Shen), was used in 15 of 29 herbal preparations. Each compound prescription contained an average of 9 ingredients (range: 2–14). The formulations of CHM were different and included tablet, capsule, oral liquid, and decoction.

### 3.4. The Effects of Interventions

#### 3.4.1. CHM versus Placebo

**Figure 2:**
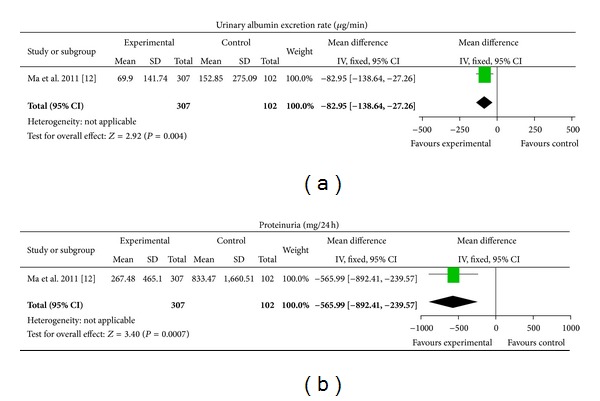
CHM versus placebo.

One trial tested Arctiin compared with placebo in patients with DN [12]. Arctiin showed significant improvement in urinary albumin excretion rate (MD −82.95 *μ*g/min, [−138.64, −27.26]) and proteinuria (MD −565.99 mg/24 h, [−892.41, −239.57]) after two months of treatment compared with placebo (Figure 2).

#### 3.4.2. CHM versus ACEI/ARB

**Figure 3:**
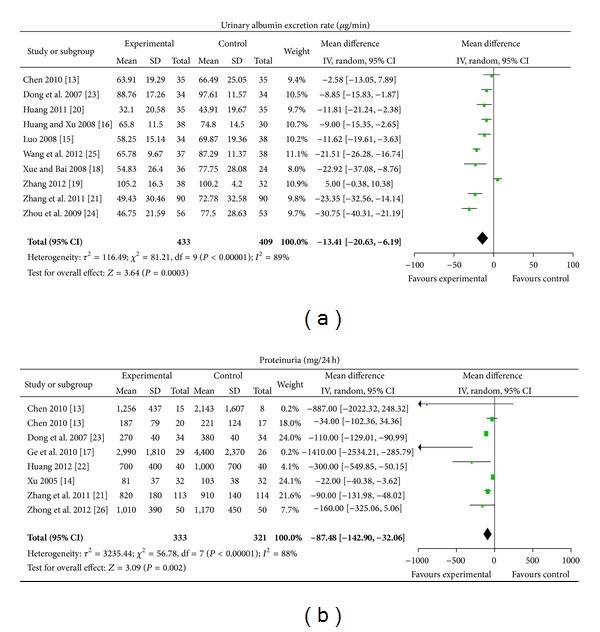
CHM versus ACEI/ARB.

14 different CHM were tested compared with ACEI/ARB [13–26], including one extract from a single herb and 13 self-composed Chinese herbal compound prescriptions. Urinary albumin excretion rate was evaluated in 10 studies and proteinuria in 8 studies. 10 trials reported significant improvement in urinary albumin excretion rate after treatment of CHM compared with ACEI/ARB (MD −13.41 *μ*g/min, [−20.63, −6.19]), with significant heterogeneity between the studies (Chi^2^ = 81.21, *I* ^2^ = 89%) (Figure 3). CHM showed significant improvement in proteinuria compared with ACEI/ARB in 8 studies (MD −87.48 mg/24 h, [−142.90, −32.06]) and there was significant heterogeneity (Chi^2^ = 56.78, *I* ^2^ = 88%) (Figure 3).

#### 3.4.3. CHM plus ACEI/ARB versus ACEI/ARB

**Figure 4:**
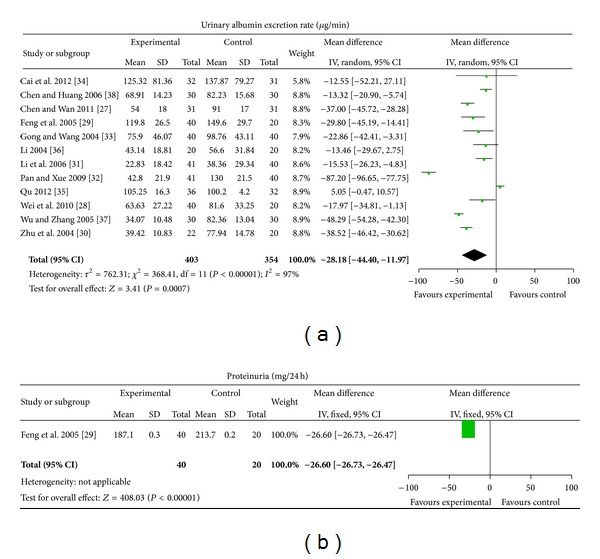
CHM plus ACEI/ARB versus no treatment plus ACEI/ARB.

*CHM plus ACEI/ARB versus No Treatment plus ACEI/ARB.* One Chinese patent medicine and 11 different self-composed Chinese herbal compound prescriptions were tested [27–38]. Urinary albumin excretion rate was evaluated in 12 studies and proteinuria in one study. CHM plus ACEI/ARB showed statistically significant improvement in urinary albumin excretion rate (MD −28.18 *μ*g/min, [−44.4, −11.97]), with significant heterogeneity between 12 studies (Chi^2^ = 368.41, *I* ^2^ = 97%) (Figure 4). One trial reported significant improvement in proteinuria after treatment of CHM plus ACEI/ARB compared with ACEI/ARB (MD −26.60 mg/24 h, [−26.73, −26.47]) (Figure 4). 

**Figure 5:**
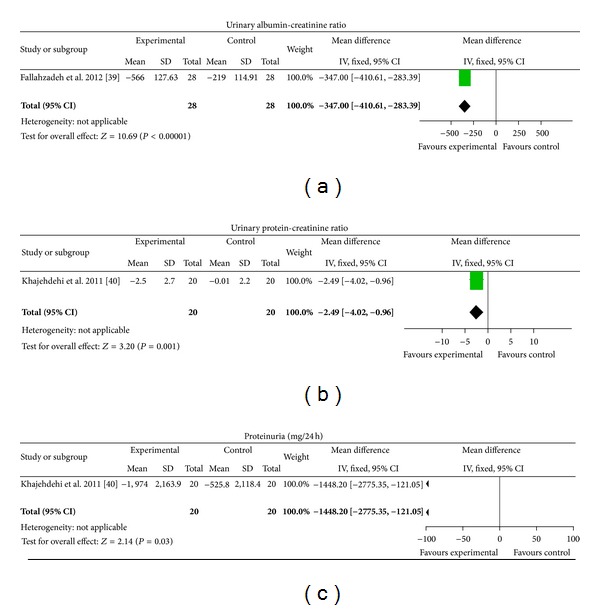
CHM plus ACEI/ARB versus placebo plus ACEI/ARB.

*CHM plus ACEI/ARB versus Placebo plus ACEI/ARB.* Two different extracts from single herbs were tested [39, 40]. Silymarin plus ACEI/ARB showed significant improvement in the change of urinary albumin-creatinine ratio from baseline (MD −347.00, [−410.61, −283.39]) compared with placebo plus ACEI/ARB (Figure 5). Turmeric plus ACEI/ARB showed significant improvement in the change of protein-creatinine ratio (MD −2.49, [−4.02, −0.96]) and proteinuria (MD −1448.20 mg/24 h, [−2775.35, −121.05]) from baseline compared with placebo plus ACEI/ARB (Figure 5).

### 3.5. Adverse Events

Fifteen trials out of 29 included trials mentioned the occurrence of adverse events [12, 13, 15, 17, 19, 24, 25, 28, 29, 32, 33, 35, 36, 39, 40]. Seven of these reported no adverse effects during herbal treatment [13, 25, 29, 32, 33, 36, 40]. Eight trials reported nonserious adverse events. Ma et al. reported that 13 out of 307 patients had experienced a variety of symptoms including abdominal pain, diarrhea, and loose stools after taking Arctiin granule [12]. These symptoms could be tolerated by patients. One patient stopped the treatment of Tripterygium glycosides due to leucopenia [17]. Among 38 patients treated with Pishen Shuangbu tang, one patient developed mild diarrhoea, and one developed dizziness [19]. The symptoms were relieved after stopping the treatment. One patient developed mild diarrhea after taking Tangshen fang [24]. Adverse effects in ACEI/ARB treated patients included dry cough, hyperkalemia, and doubling of serum creatinine [15, 17, 19, 28, 35, 39]. There was no significant difference between herbal treatment and ACEI/ARB regarding the incidence of adverse effects. No serious adverse events were reported.

## 4. Discussion

Based on the meta-analysis of 29 randomized controlled trials, CHM was tested to be more effective in reducing UAER and proteinuria compared with placebo or ACEI/ARB. Combination therapy with CHM and ACEI/ARB showed significant improvement in UAER, urinary albumin-creatinine ratio, protein-creatinine ratio, and proteinuria as compared to ACEI/ARB. It should be noted that there were no reported serious adverse events associated with CHM studied. To summarize, the results revealed that CHM is an effective and safe therapy option to treat albuminuric patients with DN.

In TCM, diabetic nephropathy referred to as an intrinsically deficient but extrinsically excessive syndrome. Deficiency of qi and yin, and excess of stasis and dampness are believed to be the main mechanism responsible for development of DN [42]. Among the included 29 RCTs, 29 different herbal preparations were tested, including four extracts from a single herb, one Chinese patent medicine, and 24 Chinese herbal compound prescriptions. Of the 24 compound prescriptions, Bushen Huoxue decoction, Pishen Shuangbu tang, and modified Liuwei Dihuang tang were prescribed based on Liuwei Dihuang tang, which has the function of nourishing the kidney yin. A total of 84 different kinds of herbs were included in 29 herbal preparations for treatment of DN. From the results of frequency distribution of categorized herbs according to their functions, herbs with qi-tonifying and yin-nourishing, blood-activating and stasis-resolving, kidney-replenishing and water-draining appeared to be most frequently prescribed for the treatment of DN. 

The pathogenesis of diabetic nephropathy is complex and not yet fully clarified. In addition to the RAS, other pathways such as oxidative stress, inflammation, and excessive production of advanced glycation end products also contribute to the development of DN [43–45]. Therefore, although use of RAS antagonists appears to slow the progression of DN development to ESRD, it does not stop or reverse the pathology. Each herbal product within the TCM formulations could have several different active ingredients to attack a disease process in manifold ways. For example, astragalus polysaccharide has prophylactic and therapeutic effects on the progress of DN by decreasing the mRNA level of NF-*κ*B in renal cortex and increasing IkB mRNA expression in rats [46]. Additionally, the antioxidative effect of *Astragalus membranaceus* as a free radical scavenger implies its protective effect in the early stage of DN [47]. *Salvia miltiorrhiza* could be applicable for the treatment of DN by reducing the serum and kidney levels of transforming growth factor *β*1 (TGF-*β*1) and the kidney levels of collagen IV, monocytes/macrophages (ED-1), and the receptor for advanced glycation end-products (RAGE) [48]. Corni Fructus has the potential to protect the animals from diabetic nephropathy by amelioration of oxidative stress and stimulation of PPAR*γ* expression [49]. These studies' results suggest that CHM can produce a potential effect of multitarget therapy, which seems appropriate in the treatment of DN caused by multiple factors.

It must be acknowledged, however, that the methodological quality of the trials evaluating the effect of CHM on DN was generally not high: 18/29 (62%) of the RCTs included in this review were scored as having mediocre methodological quality [Jadad scores = 2]. No trial was identified as a multicenter, large sample, prospective, double-blinded, controlled randomized trial. Furthermore, most of the studies did not report about allocation concealment process, which may have created potential selection bias. The possibility of publication bias in the reporting of RCTs is always of concern. Although we performed comprehensive searches and tried to avoid bias, since most of the studies were published in Chinese, there remained the possible existence of publication bias.

It is noteworthy that discrepancy in the herbal composition, drug formulation, and dose was observed between the studies, which may be the source of heterogeneity in the included RCTs. TCM formulas were composed of many herbs and the content and biological activities of these herbs can be influenced by many things, including where the herb was grown, and at what season it was harvested. Consequently, CHM for treating DN needs to equip standardized criteria for use to ensure the good reproducibility of the research result in real clinical practices. 

The results of the present review provide strong evidence of the efficacy of CHM in reducing UAER, proteinuria, urinary albumin-creatinine ratio, and protein-creatinine ratio, suggesting that CHM can be used as an alternative therapy for the treatment of DN. However, majority of included studies were scored as having mediocre methodological quality. Future clinical trials of CHM on DN need to improve methodological quality and reported well according to the CONSORT statement [50]. Hence, we conclude that further study of CHM in the treatment of DN is warranted in rigorously designed, multicentre, large-scale trials with higher quality worldwide.

## Figures and Tables

**Table 1:** Characteristics of the 29 studies included in the meta-analysis.

Author (s), year	Patients included	Men (%)	Age (years)	Albuminuria	Interventions		Treatment duration	Jadad score
					Experimental	Control		
Ma et al., 2011 [12]	409	45	56.6	Microalb Macroalb	Arctiin granule (TID)	Placebo (TID)	8 weeks	5
Chen, 2010 [13]	60	45	60.5	Microalb Macroalb	Anshen yin (TID)	Losartan (50 mg/d, QD)	12 weeks	2
Xu, 2005 [14]	64	62.5	56.2	Microalb	Baoshen tang (TID)	Benazepril (5–10 mg/d, QD)	12 weeks	2
Luo, 2008 [15]	72	54.2	56.8	Microalb	Bushen Huoxue decoction (BID)	Benazepril (10 mg/d, QD)	12 weeks	3
Huang and Xu, 2008 [16]	68	54.4	58.0	Microalb	Tangluo Tongshui decoction (BID)	Losartan (50 mg/d, QD)	8 weeks	2
Ge et al., 2010 [17]	55	56.9	51.5	Macroalb	Tripterygium glycosides (120 mg/d, TID)	Valsartan (160 mg/d, QD)	24 weeks	3
Xue and Bai, 2008 [18]	60	55.0	NA	Microalb	Liuwei Dihuang tang (BID)	Losartan (100 mg/d, QD)	12 weeks	2
Zhang, 2012 [19]	70	54.3	62.4	Microalb	Pishen Shuangbu tang (BID)	Benazepril (10 mg/d, QD)	4 weeks	2
Huang, 2011 [20]	70	52.9	56.0	Microalb	Shen an decoction (BID)	Captopril (37.5 mg/d, TID)	8 weeks	2
Zhang et al., 2011 [21]	227	NA	NA	Microalb Macroalb	Tangshen Kang capsule (TID)	Enalapril (10 mg/d, BID)	8 weeks	3
Huang, 2012 [22]	80	61.3	53.1	Macroalb	Wenshen Jianpi Huoxue tang (BID)	Benazepril (10 mg/d, QD)	8 weeks	2
Dong et al., 2007 [23]	68	57.4	55.0	Microalb	Yiqi Huoxue tang (BID)	Valsartan (80 mg/d, QD)	8 weeks	2
Zhou et al., 2009 [24]	109	38.5	54.8	Microalb	Tangshen decoction (BID)	Losartan (50 mg/d, QD)	12 weeks	3
Wang et al., 2012 [25]	75	51.3	57.2	Microalb	Yiqi Yangyin Xiaozheng Tongluo decoction (BID)	Irbesartan (150 mg/d, QD)	48 weeks	3
Zhong et al., 2012 [26]	100	53.0	48.0	Macroalb	Ziyin Zhuyang Digui tang (BID)	Benazepril (10 mg/d, QD)	12 weeks	2
Chen and Wan, 2011 [27]	62	48.4	61.6	Microalb	Qishen Yiqi drop pill (TID) Enalapril (10 mg/d, QD)	Enalapril (10 mg/d, QD)	8 weeks	2
Wei et al., 2010 [28]	60	55.0	NA	Microalb	Fufang Danpi decoction (BID) Benazepril (10 mg/d, QD)	Benazepril (10 mg/d, QD)	8 weeks	2
Feng et al., 2005 [29]	60	63.3	54.8	Microalb	Kangshen tang (BID) Benazepril (10 mg/d, QD)	Benazepril (10 mg/d, QD)	12 weeks	2
Zhu et al., 2004 [30]	42	50.0	54.8	Microalb	Pingxiao Gujing tang (BID) Benazepril (10 mg/d, QD)	Benazepril (10 mg/d, QD)	8 weeks	2
Li et al., 2006 [31]	81	49.4	50.7	Microalb	Tangshen ling decoction (BID) Telmisartan (80 mg/d, QD)	Telmisartan (80 mg/d, QD)	8 weeks	2
Pan and Xue, 2009 [32]	81	46.6	54.4	Microalb	Tangshen tang (BID) Valsartan (80 mg/d, QD)	Valsartan (80 mg/d, QD)	8 weeks	3
Gong and Wang, 2004 [33]	80	53.8	59.0	Microalb	Yangyin Yiqi decoction (BID) benazepril (10 mg/d, QD)	Benazepril (10 mg/d, QD)	8 weeks	2
Cai et al., 2012 [34]	63	63.5	41.7	Microalb	Yiqi Yangyin Huazhuo Tongluo decoction (BID) Benazepril (10 mg/d, QD)	Benazepril (10 mg/d, QD)	8 weeks	3
Qu, 2012 [35]	68	55.9	62.4	Microalb	Chunze tang (BID) Benazepril (10 mg/d, QD)	Benazepril (10 mg/d, QD)	2 weeks	2
Li, 2004 [36]	40	45.0	51.8	Microalb	Modified Liuwei Dihuang tang (BID) Enalapril (10 mg/d, QD)	Enalapril (10 mg/d, QD)	12 weeks	2
Wu and Zhang, 2005 [37]	60	43.3	59.0	Microalb	Tangshen kang (BID) Fosinopril (10 mg/d, QD)	Fosinopril (10 mg/d, QD)	8 weeks	2
Chen and Huang, 2006 [38]	60	NA	NA	Microalb	Wuchong tang (BID) Benazepril (10 mg/d, QD)	Benazepril (10 mg/d, QD)	8 weeks	3
Fallahzadeh et al., 2012 [39]	56	46.7	56.8	Macroalb	Silymarin (520 mg/d, TID) ACEI/ARB	Placebo (TID) ACEI/ARB	12 weeks	5
Khajehdehi et al., 2011 [40]	40	55	52.8	Macroalb	Turmeric (1500 mg/d, TID) ACEI/ARB	Placebo (TID) ACEI/ARB	8 weeks	4

Microalb: microalbuminuria; Macroalb: Macroalbuminuria; QD: once a day; BID: twice a day; TID: three times a day. NA: not applicable.

**Table 2:** The 14 herbs used most often for Chinese herbal preparations in the included 29 RCTs.

English herbal name (Chinese pinyin)	Number of occurrences in 29 herbal preparations	Frequency of use (%)
Astragalus (Huang Qi)	22	75.86
*Salvia miltiorrhiza* (Dan Shen)	15	51.72
Poria (Fuling)	10	34.48
Rhizoma Dioscoreae Oppositae (Shan Yao)	9	31.03
Rehmannia Root (Sheng Di Huang)	7	24.14
Fructus Macrocarpii (Shan Zhu Yu)	7	24.14
Rhizoma Polygonati Sibirici (Huang Jing)	7	24.14
Rhizoma Alismatis (Ze Xie)	7	24.14
Radix Rehmanniae preparata (Shu Di Huang)	6	20.69
Herba Leonuri Japonici (Yi Mu Cao)	6	20.69
Radix et Rhizoma Rhei Palmati (Da Huang)	6	20.69
Rhizoma Chuanxiong (Chuan Xiong)	5	17.24
Radix Codonopsis (Dang Shen)	5	17.24
Radix Pseudostellariae (Tai Zi Shen)	5	17.24

Frequency of use = number of occurrences/total number of herbal preparations.
